# Accurate Influenza Monitoring and Forecasting Using Novel Internet Data Streams: A Case Study in the Boston Metropolis

**DOI:** 10.2196/publichealth.8950

**Published:** 2018-01-09

**Authors:** Fred Sun Lu, Suqin Hou, Kristin Baltrusaitis, Manan Shah, Jure Leskovec, Rok Sosic, Jared Hawkins, John Brownstein, Giuseppe Conidi, Julia Gunn, Josh Gray, Anna Zink, Mauricio Santillana

**Affiliations:** ^1^ Computational Health Informatics Program Boston Children’s Hospital Boston, MA United States; ^2^ Harvard Chan School of Public Health Harvard University Boston, MA United States; ^3^ Department of Biostatistics Boston University School of Public Health Boston, MA United States; ^4^ Computer Science Department Stanford University Stanford, CA United States; ^5^ Chan Zuckerberg Biohub San Francisco, CA United States; ^6^ Department of Pediatrics Harvard Medical School Boston, MA United States; ^7^ Boston Public Health Commission Boston, MA United States; ^8^ athenaResearch athenahealth Watertown, MA United States

**Keywords:** epidemiology, public health, machine learning, regression analysis, influenza, human, communicable diseases, statistics, patient generated data

## Abstract

**Background:**

Influenza outbreaks pose major challenges to public health around the world, leading to thousands of deaths a year in the United States alone. Accurate systems that track influenza activity at the city level are necessary to provide actionable information that can be used for clinical, hospital, and community outbreak preparation.

**Objective:**

Although Internet-based real-time data sources such as Google searches and tweets have been successfully used to produce influenza activity estimates ahead of traditional health care–based systems at national and state levels, influenza tracking and forecasting at finer spatial resolutions, such as the city level, remain an open question. Our study aimed to present a precise, near real-time methodology capable of producing influenza estimates ahead of those collected and published by the Boston Public Health Commission (BPHC) for the Boston metropolitan area. This approach has great potential to be extended to other cities with access to similar data sources.

**Methods:**

We first tested the ability of Google searches, Twitter posts, electronic health records, and a crowd-sourced influenza reporting system to detect influenza activity in the Boston metropolis separately. We then adapted a multivariate dynamic regression method named ARGO (autoregression with general online information), designed for tracking influenza at the national level, and showed that it effectively uses the above data sources to monitor and forecast influenza at the city level 1 week ahead of the current date. Finally, we presented an ensemble-based approach capable of combining information from models based on multiple data sources to more robustly nowcast as well as forecast influenza activity in the Boston metropolitan area. The performances of our models were evaluated in an out-of-sample fashion over 4 influenza seasons within 2012-2016, as well as a holdout validation period from 2016 to 2017.

**Results:**

Our ensemble-based methods incorporating information from diverse models based on multiple data sources, including ARGO, produced the most robust and accurate results. The observed Pearson correlations between our out-of-sample flu activity estimates and those historically reported by the BPHC were 0.98 in nowcasting influenza and 0.94 in forecasting influenza 1 week ahead of the current date.

**Conclusions:**

We show that information from Internet-based data sources, when combined using an informed, robust methodology, can be effectively used as early indicators of influenza activity at fine geographic resolutions.

## Introduction

### Traditional Influenza Surveillance

Seasonal influenza is a major public health concern across the United States. Each year, over 200,000 hospitalizations from complications related to influenza infection occur nationwide, resulting in 3000 to 50,000 deaths [1]. Worldwide, up to 500,000 deaths occur annually due to influenza [2]. Vaccination is the primary prevention method [3], but other prevention and mitigation strategies are also important for reducing transmission and morbidity, including infection control procedures, early treatment, allocation of emergency department (ED) resources, and media alerts. Accurate and timely surveillance of influenza incidence is important for situation awareness and response management.

Governmental public health agencies traditionally collect information on laboratory confirmed influenza cases and reports of visits to clinics or EDs showing symptoms of influenza-like illness (ILI). ILI is symptomatically defined by the Centers for Disease Control and Prevention (CDC) as a fever greater than 100 F and cough or sore throat [4]. The CDC publishes weekly reports for national and multistate regional incidence, whereas state and city data are sometimes published by local agencies such as the Boston Public Health Commission (BPHC). These systems provide consistent historical information to track ILI levels in the US population [5,6]. However, they often involve a 1- to 2-week lag, reflecting the time needed for information to flow from laboratories and clinical databases to a centralized information system, and tend to undergo subsequent revisions. The time lag delays knowledge of current influenza activity, thus limiting the ability for timely response management. Additionally, this time lag makes it harder to predict future activity.

### Real-Time Surveillance Models

To address this issue, research teams have demonstrated the ability to monitor national and regional (collections of 5 states within the United States defined by the Department of Health and Human Services) influenza incidence in near real-time by combining various disparate sources of information. Historical flu activity shows both seasonal and short-term predictability, and models that use such autoregressive information can capture and estimate the salient features of epidemic outbreaks [7]. Rapidly updating data streams have also been found to show strong value in influenza monitoring at the national and regional levels. As early as 2006, analysis of Internet search activity has shown the potential to predict official influenza syndromic data [8]. In 2008, Google Flu Trends (GFT) launched one of the first projects to utilize Internet searches as predictors of influenza activity, eventually providing predictions from 2003 to 2015 using search volumes around the globe [9]. Although flaws have been identified in Google’s original methods and results [10], methodological improvements have shown that Internet searches are a viable way to monitor influenza [7,11-13].

Cloud-based electronic health records (EHRs) are another data source that can be obtained in near real-time [14]. Participating health care providers can report influenza cases as they occur, giving early approximations of the true infection rate. In Santillana et al [15], an ensemble approach combining these data sources outperformed any other methods in national flu predictions. In addition, traditional susceptible-infected- recovered (SIR) epidemiological models coupled with data assimilation techniques have shown strong potential in predicting influenza activity in multiple spatial resolutions [16,17]. Finally, participatory disease surveillance efforts where a collection of participants report whether they experienced ILI symptoms on a weekly basis, such as Flu Near You (FNY) in the United States, Influenzanet in Europe, and Flutracking in Australia, show promise in monitoring influenza activity in populations not frequently surveilled by health care–based surveillance systems [18-21].

### Finer Spatial Resolutions

Although significant progress in tracking and predicting influenza activity using novel data sources has been made at larger geographical scales, detection at finer spatial resolutions, such as the city level, is less well understood [7,12,14-15,22-26]. Models aiming at tracking the number of influenza-positive case rates at the city level have been developed with moderate success, including a network mechanistic model for the neighborhoods and boroughs of New York City based on the traditional SIR methodology [27]. Models combining Twitter and Google Trends data have also been tested in the same city [28], as well as in a Baltimore hospital [29].

In this paper, we demonstrate the feasibility of combining various Internet-based data sources using machine learning techniques to monitor and forecast influenza activity in the Boston metropolitan area, by extending proven methods from the national- and regional-level influenza surveillance literature to the city-level resolution. We then develop ensemble meta-predictors on these methods and show that they produce the most robust results at this geographical scale. Our methods were used to produce out-of-sample influenza estimates from 2012 to 2016 as well as out-of-sample validation on previously unseen official influenza activity data from the 2016-2017 seasons. Our contribution shows that the lessons learned from tracking influenza at broader geographical scales, such as the national and regional levels in the United States, can be adapted with success at finer spatial resolutions.

## Methods

### Data Collection

We used syndromic data collected by the BPHC as our reference for influenza activity in the metropolis. Other data sources included Google searches, Twitter posts, FNY mobile app reports, and EHRs, as described below. Data were collected from the weeks starting September 6, 2009, to May 15, 2016, and separately from the weeks starting May 22, 2016, to May 7, 2017, for the holdout set.

### Epidemiological Data

The Greater Boston area is defined using zip codes within Suffolk, Norfolk, Middlesex, Essex, and Plymouth counties. These zip codes are associated with over 90% of Boston ED visits. Limited data for ED visits from all 9 Boston acute care hospitals are sent electronically every 24 hours to the BPHC, which operates a syndromic surveillance system. Data sent include visit date, chief complaint, zip code of residence, age, gender, and ethnicity.

Our prediction target for Greater Boston was %ILI (percentage of ILI), which is calculated as the number of ED visits for ILI divided by the total number of ED visits each week. These data are updated between Tuesday and Friday with the %ILI of the previous week along with retrospective revisions of previous weeks. We used this dataset as the ground truth against which we benchmarked our predictive models. Inspection of the past 5 years of ILI activity in the Boston area, compared with US national ILI activity, shows that the peak weeks and length of outbreaks are not synchronous, and the scales (%ILI) are not necessarily comparable (Multimedia Appendix 1).

The exogenous near real-time data sources mentioned below were used as inputs to our predictive models.

#### Google Trends Data

Weekly search volumes within the Boston Designated Market Area (which has a similar size and coverage to Greater Boston) of 133 flu-related queries were obtained from the Google Trends application programming interface (API). These include query terms taken from the national influenza surveillance literature [7] as well as Boston-specific terms (all terms displayed in Multimedia Appendix 2). Each query is reported as a time series, where each weekly value represents a sample frequency out of total Google searches made during the week, scaled by an undisclosed constant. Data from the previous week are available by the following Monday. The data are left-censored, meaning that the API replaces search frequencies under some unspecified threshold with 0. To filter out the sparse data, search terms whose frequencies were over 25.1% (88/350) composed of 0s were removed, leaving 50 predictors.

#### Electronic Health Record Data

Weekly aggregated EHR data were provided by athenahealth. Although athenahealth data aggregated at the city level for Boston were not available for this study, we used state-level data as an indicator of influenza flu activity in Boston. We believe this is a suitable proxy because most of the population of Massachusetts lives in Greater Boston, which suggests that Greater Boston’s %ILI is a large subset of and likely highly correlated with the state’s %ILI. Three time series at the Massachusetts’ level were used as our input variables: “influenza visit counts,” “ILI visit counts,” and “unspecified viral or ILI visit counts.” A fourth time series, “total patient visit counts,” was used to convert the case counts into rates. Reports from the previous week are available on the following Monday. Detailed information on EHR data from athenahealth is provided in Santillana et al [14].

To convert the 3 influenza-related case counts into frequencies, they were each divided with a 2-year moving average of the weekly total patient visits to construct smoothed rate variables. The justification for this approach is provided in Multimedia Appendix 2.

#### Flu Near You Data

FNY is an Internet-based participatory disease surveillance system that allows volunteers in the United States and Canada to report their health information during the previous week and in real-time, using a brief survey. The system collects and publishes symptom data on its website on a weekly basis and offers an interface to compare its data with data from the CDC sentinel influenza network [18]. Data for the previous week are available the following Monday. FNY participants located in the Greater Boston area were identified using the zip code provided at registration. Raw FNY %ILI for Boston was calculated by dividing the number of participants reporting ILI in a given week by the total number of FNY participant reports in that same week.

#### Twitter Data

We used the GNIP Historical Powertrack service to collect all tweets from April 15, 2015, to March 24, 2017, that were geocoded (using the GNIP location field) within a 25-mile radius from Boston (defined as 42.358056, −71.063611), the maximum radius supported by GNIP. The definition of Greater Boston used in this study is approximately the same radius. A subset of tweets was extracted from the Twitter dataset according to criteria specified by a generated list of key influenza-related terms and phrases. Initialized with a set of common hashtags related to disease (including #sick and #flu), the list was expanded based on linguistic term associations identified in disease-related tweets to include terms such as #stomachache and #nyquil.

### Models

We adapted a variety of models from the influenza surveillance literature to answer the following 2 questions: (1) What are the data sources that best track influenza activity as reported by the BPHC? and (2) What are the methodologies that best estimate the influenza activity by combining the data sources identified in (1)?

The models fall into 2 categories: single source variable analyses to investigate the value of a specific dataset in tracking %ILI and multisource analyses to inspect the value of combining disparate information sources for tracking %ILI. Motivated by the analyses presented in Yang et al for national influenza tracking [30], most of our models combine 52-week autoregressive components with terms from real-time Internet-based data in a multivariate linear regression with L1 regularization (LASSO). The initial regression model was trained using the first 2 years of data (104 weeks), and subsequent models were retrained each week using the 2-year sliding window (ie, most recent 104 weeks of data). Following the convention in [30], these models are indicated as ARGO (autoregression with general online information).

Because exogenous data for each week are available by the following Monday, whereas the official BPHC %ILI is published by the following Friday, we have 2 useful estimation targets: (1) a nowcast of %ILI for the week that just ended (concurrent with the exogenous data) and (2) a forecast of %ILI over the coming week (1 week ahead of the exogenous data). Predictions on the forecast horizon were produced by retraining the models from the nowcast horizon with the %ILI targets shifted 1 week forward.

#### Models on Single Data Sources

##### Endogenous Model

###### AR52

An autoregressive baseline model was constructed to evaluate the benefit of using only past values of the BPHC %ILI time series to estimate the current %ILI. To predict the %ILI in a given week, the %ILI of the previous 52 weeks was used as the independent variables in a LASSO regression.

##### Exogenous Models

###### ARGO(FNY)

The raw FNY rate at time *t* was combined with 52 autoregressive terms in a LASSO regression.

###### ARGO(Google)

We constructed the model presented in [7], using the Google Trends search frequencies and 52 autoregressive terms in a LASSO regression.

###### ARGO(athena)

As in [14], athenahealth rates from the 3 most recently available weeks were combined for each week’s prediction, resulting in a stack of 9 variables. These weeks are denoted as “ *t*-1,” “ *t*-2,” and “ *t*-3” in our analysis. The model combines the 9 variables at time *t* with 52 autoregressive terms in a LASSO regression.

###### Twitter

The modeling approach involved developing a multistage pipeline framework described in detail in [31]. Initially, a list of flu-related tweets was extracted as described in the Data section. We subsequently clustered each relevant tweet within its hashtag corpus according to the calculated term frequency–inverse document frequency vectors [32], and we classified a random subset of tweets within each cluster into 3 categories—self reporting, non-self-reporting, and spam—according to a second set of engineered linguistic attributes. Clusters with large proportions of non-self-reporting and spam tweets were subsequently eliminated, with the remaining tweets and associated timestamps forming a daily frequency distribution corresponding to %ILI over time. The results were finally aggregated at the weekly level and scaled to the BPHC %ILI. Because Twitter data were available for a period of less than 2 years, we did not include Twitter in our ARGO models.

###### ARGO(athena+Google+FNY)

The athenahealth rates, Google Trends search frequencies, and raw FNY rate were combined with 52 autoregressive terms in a modified LASSO regression with grouped regularization as in [30]. The model includes additional processing and hyper-parameter settings, details of which are presented in Multimedia Appendix 3.

###### Ensemble

Finally, we developed a meta-predictor on a layer of 7 input models, including most of those previously defined. The flu estimates of these input models were combined based on the historical performances of the models. In the nowcast horizon, a performance-adjusted median on the outputs of the individual models was selected as the ensemble meta-predictor. In the forecast horizon, a performance-adjusted LASSO regression was selected as the ensemble meta-predictor.

A detailed description and comparison of all models, including ensembles, are presented in Multimedia Appendix 4.

All experiments were conducted in Python 2.7 (Python Software Foundation) using scikit-learn version 0.18.1 [33].

#### Models Combining Multiple Datasets

### Comparative Analyses

Model performance was evaluated using 5 metrics: root mean square error (RMSE), mean absolute error (MAE), mean absolute percentage error (MAPE), Pearson correlation coefficient (CORR), and correlation of increment (COI). For an estimation *ŷ* of the official %ILI *y*, the definitions are as follows:

RMSE = [ ( 1 / *n* ) ∑_t=1…__n_ ( *ŷ*_t_ – *y*_t_ ) ^2^ ] ^1/2^

MAE = ( 1 / *n* ) ∑_t=1…__n_ | *ŷ*_t_ – *y*_t_ |

MAPE = ( 1 / *n* ) ∑_t=1…__n_ | *ŷ*_t_ – *y*_t_ | / *y*_t_

COI = CORR ( *ŷ*_t_ – *ŷ*_t-1_ , *y*_t_ – *y*_t-1_ )

For guidance, a method is more accurate when the prediction errors (RMSE, MAE, and MAPE) are smaller and closer to 0. The LASSO objective minimizes RMSE, so this metric will be our primary way to assess model accuracy. A method tracks the movement of the flu activity better when the correlation values (CORR and COI) are closer to 1.

Metrics were computed between each model’s predictions and the official BPHC %ILI over the entire test period (September 2, 2012, to May 15, 2016), as well as for each influenza season (week 40 to week 20 of the next year) including the holdout set.

The following 2 additional benchmarks were constructed to establish a baseline for comparison between all models:

1. A naive model that uses the %ILI from the previous week as the prediction for the current week
2. GFT influenza activity estimates from September 5, 2010, to August 9, 2015, accessed on December 2016, from [9].

Because Google reported values as intensities between 0 and 1 without a clear scaling constant, the data were linearly rescaled to fit BPHC %ILI using the same initial training set as the above models (September 5, 2010, to August 26, 2012).

## Results

Out-of-sample weekly estimates of Greater Boston ILI activity from all models were produced retrospectively for the period starting from September 2, 2012, to May 15, 2016. After the 2016-2017 flu season, previously unseen BPHC %ILI data from May 22, 2016, to May 7, 2017, were used to validate model performances.

### Single Data Source Evaluation

**Figure 1:**
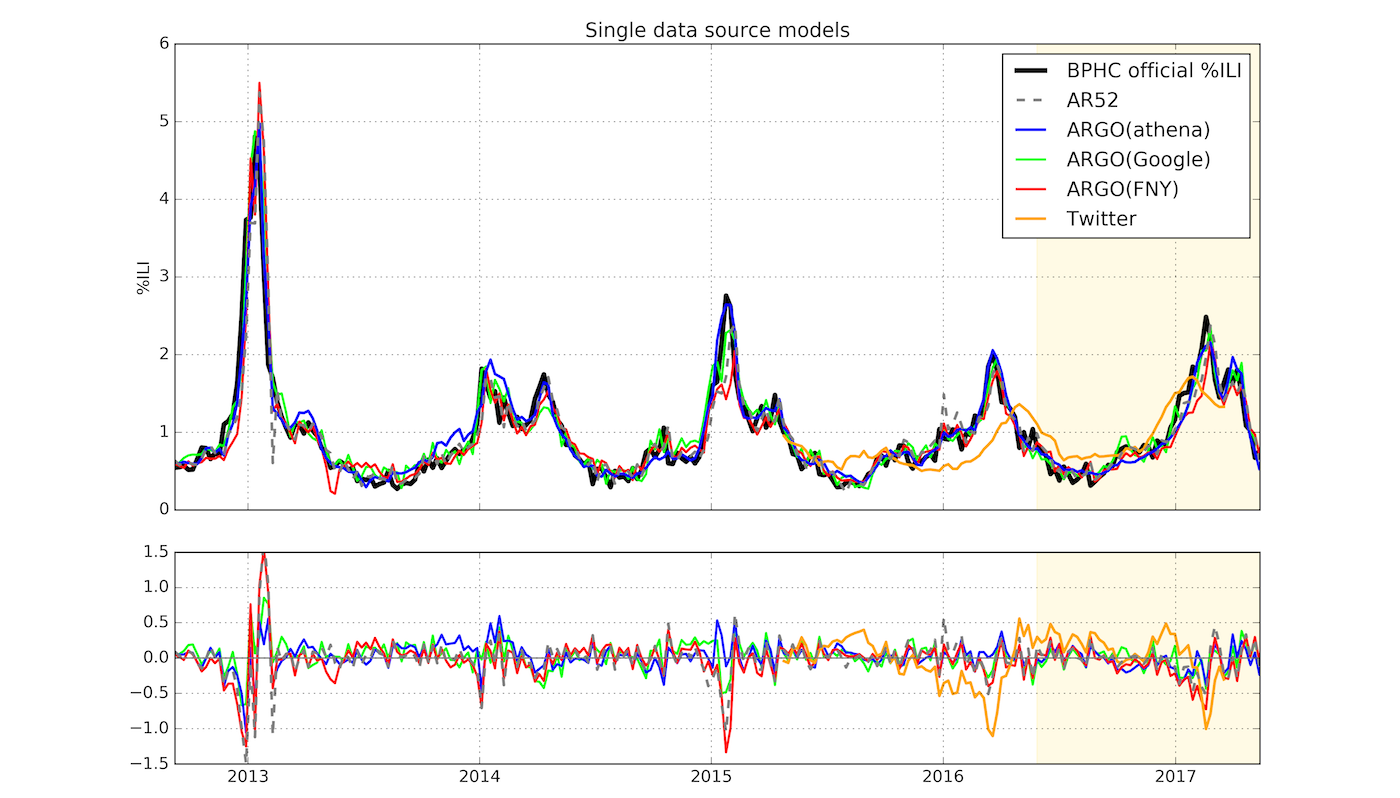
Retrospective nowcasts from single data source models are shown, compared with Boston Public Health Commission’s official percentage of influenza-like illness (BPHC official %ILI) (black), over the entire study period (September 2, 2012, to May 7, 2017). The gold section indicates the holdout period from May 22, 2016, to May 7, 2017. The bottom panel shows the corresponding errors of each model compared with the official %ILI (ARGO: autoregression with general online information; FNY: Flu Near You).

Table 1 shows the metrics calculated by comparing retrospective estimates from all models built with a single exogenous dataset against BPHC %ILI, over 5 flu seasons. ARGO(athena) is the best performing model in this category, both overall and across the majority of flu seasons. For example, ARGO(athena) yields a 5% (0.011/0.206) lower RMSE than the nearest competitor ARGO(Google), 36% (0.108/0.303) lower error than AR52, and 27% (0.071/0.266) lower error than the naive approach. As shown in Figure 1, ARGO(athena) tends to capture peaks of ILI activity more accurately than the other models.

Because each ARGO model in Table 1 combines an exogenous dataset with AR52, comparing each model with AR52 indicates how much predictive value the dataset adds to the historical ILI time series. Both athenahealth and Google Trends datasets show a marked improvement over a simple AR52 model, indicating that they contain valuable information for influenza monitoring. Both models also demonstrate reduced error (RMSE, MAE, and MAPE) compared with the GFT benchmark in seasons where GFT was available. ARGO(FNY) performs about the same as AR52 overall, indicating that FNY may not necessarily track the BPHC %ILI. It is important to highlight that although the Twitter %ILI estimates perform worse than all other models, this approach was not dynamically trained with AR52 information, due to the short period when these values were produced. Twitter %ILI estimates nevertheless show a similar pattern of peaks and dips compared with the BPHC %ILI (Figure 1).

### Multiple Data Sources Evaluation

In Table 2, the performance of the best single-dataset model, ARGO(athena), is compared with the performance of the multi-dataset models for the nowcast horizon. Over the entire period and for all flu seasons besides 2015-16, ARGO(athena+Google+FNY) shows a decrease in error and increase in correlation compared with ARGO(athena). In particular, it achieves a 15% (0.03/0.195) decrease in RMSE and a 20% (0.109/0.547) increase in COI compared with ARGO(athena), significantly improving on the performance of the single-dataset approach. A similar pattern is present in the one week ahead forecast horizon, with significantly better performance over the entire period except for the 2014-15 and 2015-16 seasons (Table 3). With a 25% (0.08/0.325) decrease in RMSE and a 23% (0.101/0.432) increase in COI compared with ARGO(athena), the multi-dataset model again provides a distinct improvement.

The comparison between ARGO(athena+Google+FNY) and ARGO(athena) shows that models combining multiple data sources generally perform better than the best dataset alone, consistent with previous findings on influenza prediction at the US national level [15,30]. However, the superiority of ARGO(athena+Google+FNY) is not consistent over all seasons. As shown in Multimedia Appendix 4, when compared with the full array of models we tested, ARGO(athena+Google+FNY) underperforms in not only the seasons previously mentioned where it loses to ARGO(athena) but also in the 2013-14 influenza season. In other words, even though ARGO(athena+Google+FNY) is overall stronger than the other models discussed, its results in any given season could be significantly worse than the best-performing model of that season. Similarly, the other models (non-ensembles) exhibit variations in performance over time, with none consistently performing at the top.

### Ensemble Modeling Approach Evaluation

**Figure 2:**
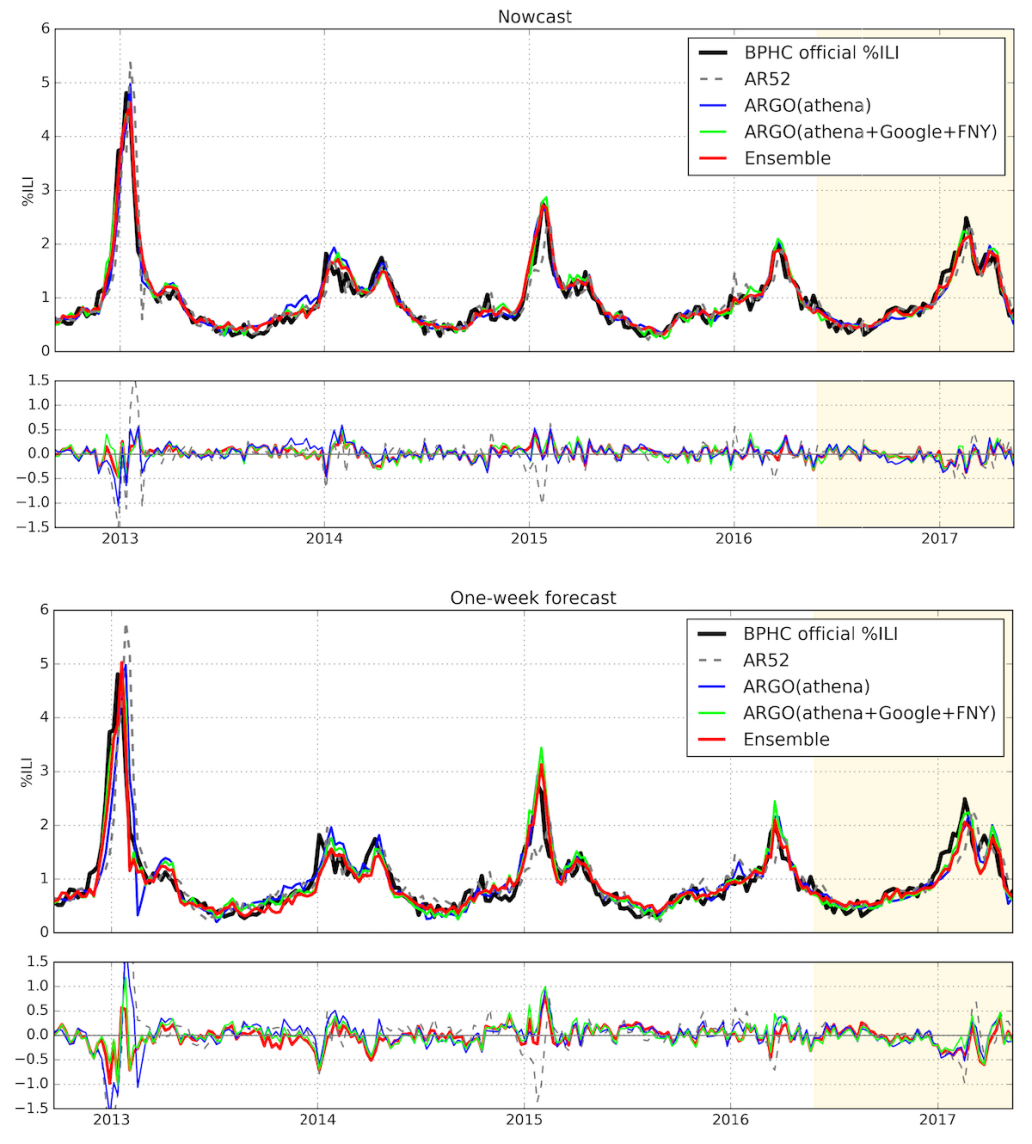
Estimations with multiple data source models over the entire study period (September 2, 2012, to May 7, 2017), with corresponding errors for each model compared with Boston Public Health Commission percentage of influenza-like illness (BPHC official %ILI). The gold section indicates the holdout period from May 22, 2016, to May 7, 2017. Predictions are shown separately for the nowcast horizon (top) and the forecast horizon (bottom) (ARGO: autoregression with general online information; FNY: Flu Near You).

To develop a more robust and consistent set of influenza estimates, we trained an ensemble meta-predictor that takes predictions from all the above models and combines them into a single prediction. As shown in Tables 2 and 3, our ensembles achieve the best overall performance in every metric, in both nowcast and forecast horizons. In the nowcast, the ensemble is consistently the strongest model, with the lowest RMSE and highest correlation in 4 out of 5 seasons. In the forecast, the meta-predictor is less dominant over ARGO(athena+Google+FNY), but still has the advantage of consistency: even when it is not the strongest model over a season, it is never far from the best performance. This is illustrated in Multimedia Appendix 4, where the ensembles achieve top 2 performances over all influenza seasons more consistently than any other model in the input layer. Figure 2 confirms this consistency by showing that the ensemble curve accurately predicts the magnitude of peaks in each influenza season, with less prediction error than the other models.

We found that different ensemble methods performed better at each time horizon. The results of 4 different meta-predictors are shown in Multimedia Appendix 4. The performance-adjusted median showed the best performance for the nowcast, and LASSO showed the best performance for the forecast.

In multiple seasons including 2016-17, the predicted nowcast peak occurs slightly later than the observed %ILI peak (Figure 1), which likely occurs because of the autoregressive contribution in the input variables for each model. This delay becomes more significant when predicting the 1-week forecast, as shown in the bottom panel of Figure 2. As noted in Yang et al [7], a trade-off occurs between robustness and responsiveness when using ARGO, where robustness refers to avoiding large errors in any given week and responsiveness refers to predicting the gold standard without delay. The top panel of Figure 2 shows that the presence of this lag in the nowcast is mitigated when using our ensemble approach, improving responsiveness while preserving robustness.

**Table 1:** Comparison of single data source models for nowcasting Boston Public Health Commission’s percentage of influenza-like illness over the assessment period (September 2, 2012, to May 7, 2017). Each flu season starts on week 40 and ends on week 20 of the next year.

Model^a^		Whole period	Flu seasons				Holdout
		2012-16	2012-13	2013-14	2014-15	2015-16	2016-17
**Root mean square error**							
	AR52	0.303	0.577	0.199	0.305	0.217	0.229
	ARGO(athena)^b^	*0.195*	*0.306*	0.229	*0.192*	*0.133*	*0.182*
	ARGO(Google)	0.206	0.312	*0.194*	0.247	0.161	0.188
	ARGO(FNY)^c^	0.299	0.552	0.204	0.343	0.172	0.280
	Twitter	—	—	—	0.162	0.427	0.351
	GFT^d^	—	0.352	0.271	0.284	—	—
	Naive	0.266	0.481	0.208	0.280	0.202	0.219
**Mean absolute error**							
	AR52	0.180	0.345	*0.146*	0.218	0.176	0.176
	ARGO(athena)	*0.137*	*0.205*	0.189	*0.136*	*0.102*	0.154
	ARGO(Google)	0.150	0.206	0.155	0.213	0.131	*0.153*
	ARGO(FNY)	0.182	0.362	0.153	0.221	0.140	0.213
	Twitter	—	—	—	0.136	0.338	0.251
	GFT	—	0.294	0.225	0.227	—	—
	Naive	0.167	0.290	0.165	0.213	0.158	0.168
**Mean absolute percentage error**							
	AR52	0.184	0.188	*0.137*	0.185	0.188	0.130
	ARGO(athena)	*0.163*	*0.128*	0.193	*0.124*	*0.110*	0.129
	ARGO(Google)	0.179	0.134	0.152	0.209	0.146	*0.125*
	ARGO(FNY)	0.192	0.210	0.139	0.186	0.143	0.157
	Twitter	—	—	—	0.212	0.308	0.189
	GFT	—	0.308	0.221	0.228	—	—
	Naive	0.172	0.169	0.152	0.188	0.162	0.130
**Pearson correlation coefficient**							
	AR52	0.898	0.882	0.846	0.834	0.806	0.898
	ARGO(athena)	*0.959*	0.964	0.843	*0.950*	*0.943*	*0.949*
	ARGO(Google)	0.956	0.968	*0.856*	0.910	0.896	0.930
	ARGO(FNY)	0.901	0.909	0.845	0.824	0.886	0.879
	Twitter	—	—	—	0.888	0.416	0.759
	GFT	—	*0.974*	0.785	0.921	—	—
	Naive	0.922	0.912	0.846	0.868	0.848	0.906
**Correlation of increment**							
	AR52	0.222	0.359	−0.105	0.115	−0.048	0.222
	ARGO(athena)	*0.547*	0.657	0.220	0.483	*0.486*	*0.515*
	ARGO(Google)	0.546	0.730	*0.399*	0.284	0.267	0.417
	ARGO(FNY)	0.252	0.387	−0.056	−0.025	0.122	0.253
	Twitter	—	—	—	−0.481	−0.291	0.095
	GFT	—	*0.892*	0.281	*0.575*	—	—
	Naive	0.291	0.480	-0.100	0.280	−0.070	0.193

^a^The best performance within each season and metric is italicized. Results for each model are shown where available.

^b^ARGO: autoregression with general online information.

^c^FNY: Flu Near You.

^d^GFT: Google Flu Trends.

**Table 2:** Comparison of models using multiple data sources for nowcasting Boston Public Health Commission’s percentage of influenza-like illness over the study period (September 2, 2012, to May 7, 2017). ARGO(athena) and the naive model are included as benchmarks for comparison.

Model^a^		Whole period	Flu seasons				Holdout
		2012-16	2012-13	2013-14	2014-15	2015-16	2016-17
**Root mean square error**							
	ARGO(athena)^b^	0.195	0.306	0.229	0.192	*0.133*	0.182
	ARGO(athena+Google+FNY)^c^	0.165	0.199	0.192	0.189	0.168	0.156
	Ensemble	*0.151*	*0.193*	*0.170*	*0.176*	0.139	*0.150*
	Naive	0.266	0.481	0.208	0.280	0.202	0.219
**Mean absolute error**							
	ARGO(athena)	0.137	0.205	0.189	0.136	*0.102*	0.154
	ARGO(athena+Google+FNY)	0.124	0.146	0.144	0.154	0.128	0.131
	Ensemble	*0.112*	*0.140*	*0.131*	*0.135*	0.106	*0.118*
	Naive	0.167	0.290	0.165	0.213	0.158	0.168
**Mean absolute percentage error**							
	ARGO(athena)	0.163	0.128	0.193	*0.124*	*0.110*	0.129
	ARGO(athena+Google+FNY)	0.154	0.112	0.136	0.153	0.142	0.104
	Ensemble	*0.140*	*0.100*	*0.123*	0.132	0.118	*0.093*
	Naive	0.172	0.169	0.152	0.188	0.162	0.130
**Pearson correlation coefficient**							
	ARGO(athena)	0.959	0.964	0.843	0.950	*0.943*	0.949
	ARGO(athena+Google+FNY)	0.972	0.985	0.861	*0.964*	0.916	0.957
	Ensemble	*0.976*	*0.986*	*0.890*	*0.964*	0.928	*0.958*
	Naive	0.922	0.912	0.846	0.868	0.848	0.906
**Correlation of increment**							
	ARGO(athena)	0.547	0.657	0.220	0.483	*0.486*	0.515
	ARGO(athena+Google+FNY)	0.656	0.807	0.419	*0.660*	0.312	*0.620*
	Ensemble	*0.689*	*0.827*	*0.447*	0.633	0.357	0.565
	Naive	0.291	0.480	−0.100	0.280	−0.070	0.193

^a^The best performance within each season and metric is italicized.

^b^ARGO: autoregression with general online information.

^c^FNY: Flu Near You.

**Table 3:** Comparison of models using multiple data sources for forecasting Boston Public Health Commission’s percentage of influenza-like illness, over the study period (September 2, 2012, to May 7, 2017). ARGO(athena) and the naive model are included as benchmarks for comparison.

Model^a^		Whole period	Flu seasons				Holdout
		2012-16	2012-13	2013-14	2014-15	2015-16	2016-17
**Root mean square error**							
	ARGO(athena)^b^	0.325	0.647	0.249	0.260	0.188	0.261
	ARGO(athena+Google+FNY)^c^	0.245	0.367	*0.221*	0.314	0.190	*0.240*
	Ensemble	*0.222*	*0.348*	0.237	*0.251*	*0.155*	0.251
	Naive	0.428	0.827	0.276	0.447	0.271	0.340
**Mean absolute error**							
	ARGO(athena)	0.203	0.432	0.200	*0.184*	0.156	0.221
	ARGO(athena+Google+FNY)	0.169	0.247	*0.161*	0.225	0.151	*0.189*
	Ensemble	*0.157*	*0.245*	0.171	0.202	*0.123*	0.198
	Naive	0.252	0.528	0.203	0.329	0.201	0.251
**Mean absolute percentage error**							
	ARGO(athena)	0.217	0.254	0.186	*0.168*	0.163	0.175
	ARGO(athena+Google+FNY)	0.192	0.167	*0.142*	0.214	0.147	0.149
	Ensemble	*0.180*	*0.160*	0.155	0.198	*0.130*	*0.144*
	Naive	0.238	0.308	0.169	0.272	0.195	0.204
**Pearson correlation coefficient**							
	ARGO(athena)	0.887	0.842	0.785	*0.933*	0.898	0.891
	ARGO(athena+Google+FNY)	0.938	0.949	0.826	0.922	0.903	0.910
	Ensemble	*0.944*	*0.956*	*0.847*	0.916	*0.906*	*0.913*
	Naive	0.799	0.739	0.744	0.663	0.728	0.775
**Correlation of increment**							
	ARGO(athena)	0.432	0.452	0.259	*0.624*	*0.312*	0.472
	ARGO(athena+Google+FNY)	0.533	0.621	*0.451*	0.508	0.285	0.477
	Ensemble	*0.573*	*0.682*	0.441	0.515	0.286	*0.510*
	naive	0.102	0.212	0.174	−0.181	0.043	−0.065

^a^The best performance within each season and metric is italicized.

^b^ARGO: autoregression with general online information.

^c^FNY: Flu Near You.

### Relevance of Different Data Sources

**Figure 3:**
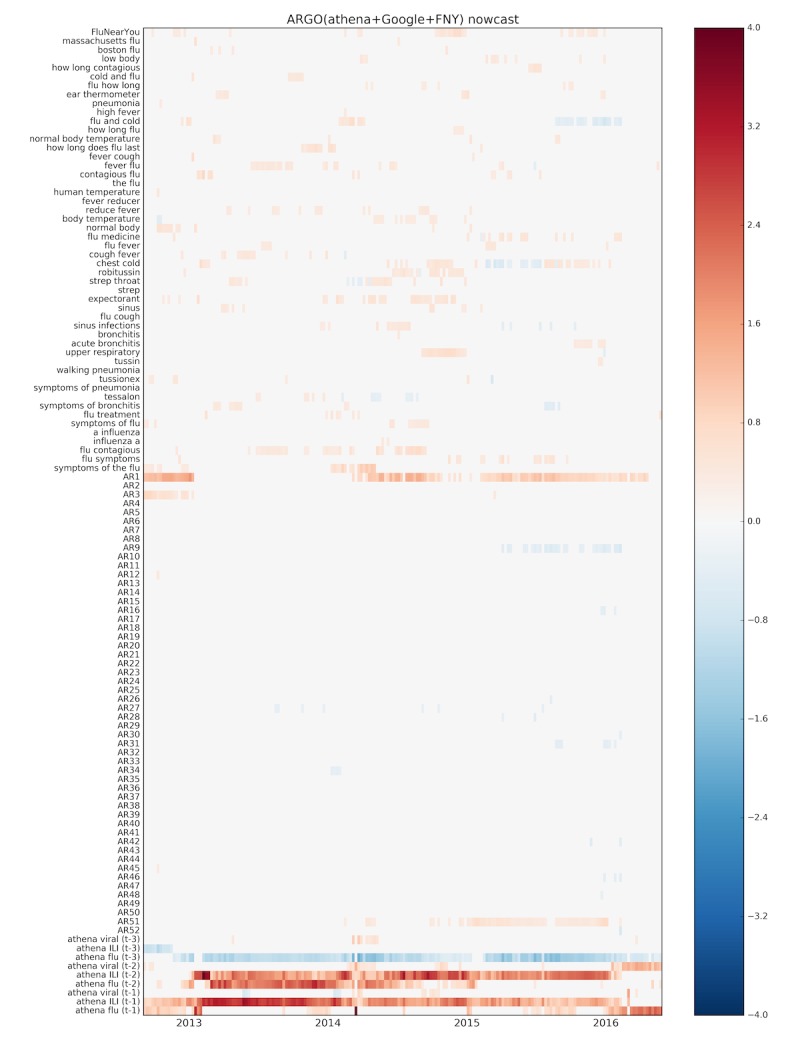
Heatmap of input variable coefficients for ARGO(athena+Google+FNY) from September 2, 2012, to May 15, 2016, for the nowcast horizon (ARGO: autoregression with general online information).

**Figure 4:**
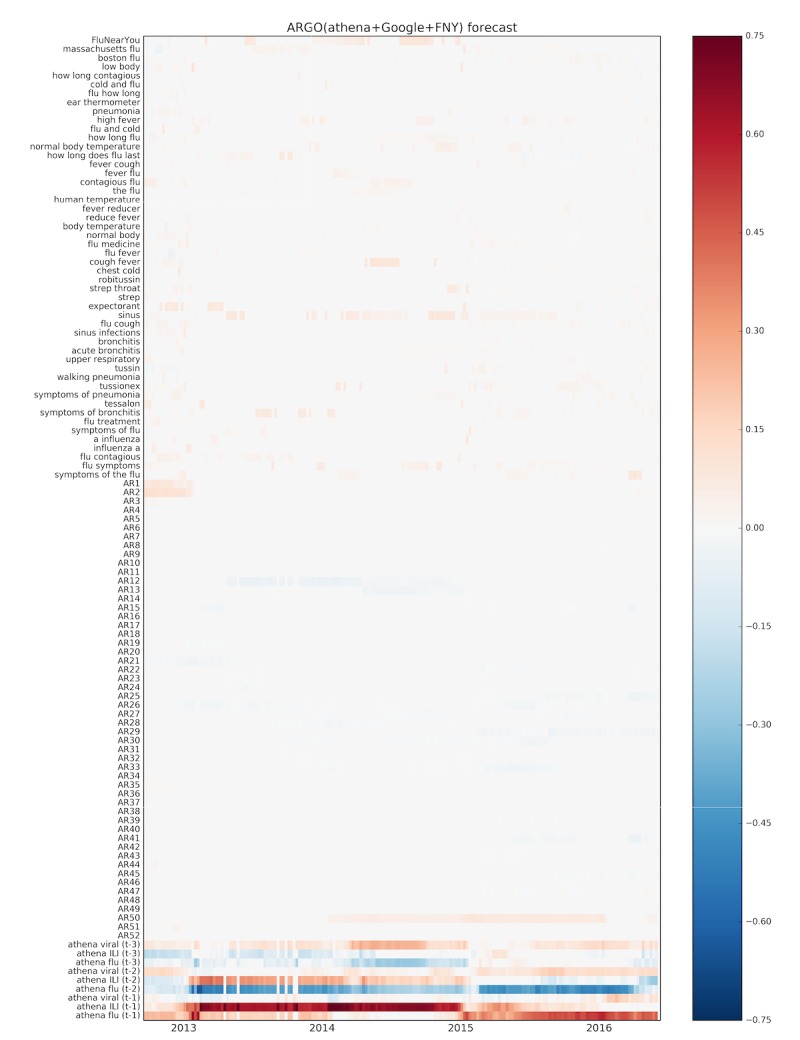
Heatmap of input variable coefficients for ARGO(athena+Google+FNY) from September 2, 2012, to May 15, 2016, for the 1-week forecast horizon (ARGO: autoregression with general online information).

To understand the predictive power of each data source when used together as input, we displayed the weekly coefficient values associated with each (normalized) input variable in the ARGO(athena+Google+FNY) model, over time, in heatmaps (Figures 3 and 4). It can be seen that the state-level athenahealth variables have the strongest signal in both nowcast and forecast horizons, suggesting that information from EHRs is a strong predictor of metropolitan level influenza. Although most of the long-term autoregressive terms show little to no signal, the most recent ILI value is predictive for the nowcast horizon. Finally, the selection of Google Trends variables and FNY by LASSO appears to be fairly scattered, with a few terms such as “chest cold,” “flu contagious,” and “sinus” appearing more consistently. Interestingly, FNY reports show a stronger signal in the forecast time horizon, suggesting that perhaps early self-reporting of symptoms correlates with later ED visits.

**Table 4:** Efficiency improvement of ensemble method with 95% confidence intervals over the period of September 2, 2012, to May 15, 2016, using the stationary block bootstrap with mean length 52 weeks.

Model		Mean^a^	95% CI
**Nowcast**			
	AR52	4.03	2.11-6.83
	athena	1.91	1.14-3.01
	Google	2.16	1.46-2.78
	athena+Google	1.51	1.25-1.77
	ARGO(athena)^b^	1.67	1.31-2.18
	ARGO(Google)	1.87	1.57-2.30
	ARGO(athena+Google+FNY)^c^	1.20	1.10-1.29
**Forecast**			
	AR52	4.55	1.98-7.16
	athena	2.37	1.54-3.14
	Google	2.78	1.98-3.63
	athena+Google	2.21	1.20-3.24
	ARGO(athena)	2.14	1.20-3.08
	ARGO(Google)	6.09	2.12-10.27
	ARGO(athena+Google+FNY)	1.21	1.03-1.35

^a^Mean values of the error for each methodology are displayed as multiples of the error associated to the best ensemble approach (for which the efficiency is assigned to be 1).

^b^ARGO: autoregression with general online information.

^c^FNY: Flu Near You.

### Statistical Significance Test

Using the stationary block bootstrap for time series [34], we calculated the mean square error reduction of the ensemble method compared with all other models. The efficiency metrics and corresponding 95% confidence intervals are provided in Table 4. In the nowcast time horizon, our ensemble method shows a four-fold error reduction when compared with an autoregressive model, and a 17% (0.2/1.2) error reduction over the best multi-dataset model ARGO(athena+Google+FNY). The confidence intervals confirm the statistical significance of these results. Our ensemble method shows similar improvements in the forecast horizon as well.

## Discussion

### Indicators of Influenza Activity

Robust estimates of influenza activity in a population are desirable to monitor and prepare for unusual events. However, different sectors of the population in a local area behave differently, and thus, the incidence and dynamics of the spread of flu cannot be captured by a single system. Multiple influenza surveillance systems may provide valuable complementary information representing activity in multiple interacting populations within an area. For example, syndromic surveillance systems, such as the one set up by BPHC, provide the number of people seeking emergency care with ILI symptoms. We used BPHC %ILI as our reference for syndromic ILI in this paper, because it has been well established and consistently reporting for several years. Previous analysis of the coordination between the BPHC ILI syndrome coding and official lab results has shown a correlation of 0.84, *P*<0.001, with Boston viral isolate data [35]. On the other hand, crowd-sourced systems such as FNY, where only 35% of self-reported sick users visit a doctor [19], may help us understand influenza activity in populations that may not seek medical treatment or at times when weather activity, such as snowstorms, limits access to health care systems. The network of outpatient providers served by athenahealth characterizes yet another sector of the population. As such, there is no gold standard of influenza activity, and surveillance systems should be compared to identify if upward or downward trends are observed across them.

### Analysis of Our Findings

Our study shows that novel influenza surveillance approaches that leverage information from Internet search engines, Twitter posts, self-reporting crowd-sourced influenza reports, and EHRs can monitor and forecast influenza activity as reported by a well-established metropolitan surveillance system, in near real-time. In terms of tracking the BPHC %ILI, our findings show that Google search frequency data and EHR information have strong predictive power. This confirms that these 2 data sources are valuable indicators of ILI activity, not only at the national and regional scales but also at spatial resolutions as small as the metropolitan level. Machine learning models based on these data sources outperform autoregressive models based solely on past values of the BPHC %ILI time series, achieving lower error and higher correlations. Combining the above information and other data sources, such as FNY, using methods that dynamically learn from the past, results in strong performances in both monitoring and forecasting influenza activity.

Compared with previous studies at the national level, combining all data sources in a single model, specifically ARGO(athena+Google+FNY), does not show the same degree of precision or consistency at the metropolitan level. We believe that the finer spatial resolution of Boston compared with national and regional levels may be a limiting factor for the quality of some of the exogenous datasets used in this study, namely, Google Trends, Twitter, and FNY. As noted in previous studies, as we zoom in on finer spatial resolutions, we found that (1) Google search frequencies are more susceptible to noise from people who may search for flu-related terms but are not infected by the flu [30,36], (2) the accuracy of Twitter-based disease surveillance decreases as it becomes difficult to capture enough related Twitter posts to extrapolate an %ILI curve [37,38], and (3) FNY may have too few participants to infer meaningful population-wide flu incidence estimates, as Boston receives only around 300 reports per week. This can result in variables having inconsistent predictive strength over time and different seasons. Because ARGO selects variables for a week’s regression based on predictive strength over the past 104 weeks, it may give worse estimations when, for example, a variable that performed strongly over the training set starts to perform poorly in the current season.

Ensemble approaches show promise for achieving robust results in situations where the performance of a single method over time is not robust. By allowing contributions from a group of different models, individual fluctuations of accuracy tend to be smoothed out. In an ideal situation where each model is valid but biased differently, the median is a robust way to lend equal weight to all models. In our case, where there are clear differences in overall predictive strength between models, we needed to apply a performance-based adjustment. Our proposed ensemble for the nowcast dynamically rewards the most accurate model and penalizes the least accurate model in an out-of-sample fashion each week. In the forecast horizon, however, the median approach was unsuccessful, suggesting that there are systematic biases (eg, all of the models overpredict in some weeks) that the median fails to correct for. However, a regression-based methodology, which was the best method for the forecast horizon, allows systematic error to be modeled and incorporated into the model, at the cost of being more susceptible to the original issue of overfitting on inconsistencies over the training set. (In this case, rather than a data source being inconsistent over time, it is the model produced on that data source that is inconsistent.) These trade-offs may explain why different methods were successful at each estimation horizon.

Models utilizing Google Trends information performed especially well compared with the naive method in the 2012-13 season but poorly in the subsequent season. The 2012-13 season notably featured a large outbreak of influenza, which led the naive and AR52 methods to perform especially poorly as their predictions tend to be lagged. In such a situation, Google Trends data can improve the model by adding responsiveness. This situation is reflected in the nowcast heatmap (Figure 3), where the signal from AR1 disappears at the beginning of 2013, as the big influenza peak was occurring. At around the same time, athenahealth signal increased and remained consistent until 2016, suggesting that athenahealth increased its strength as a predictor of BHCP %ILI. This is supported by Table 1, where ARGO(athena) shows a steady decrease in RMSE from the 2012-2013 season until the 2015-2016 season. The overall improvement suggests that as athenahealth becomes further established, its predictive accuracy may continue to rise.

### Using Influenza-Like Illness Incidence as the Target Variable

We chose to estimate %ILI visit rates as our target rather than attempt to infer diagnosed influenza incidence. On one hand, confirmed influenza case counts are useful for specifying transmission dynamic models and virology analysis. On the other hand, %ILI incidence serves a practical and actionable role by directly predicting the quantity of ILI visits that health care facilities may need to be prepared for. Preparedness is an increasingly significant task for public health and requires an epidemiology framework beyond outbreak detection, because influenza activity is not uniform across geographic areas and downward trends are equally important as upward trends for decision making. ILI nowcasting and forecasting at the local level can improve the timeliness, efficiency, and effectiveness of response and control measures. Examples include planning (long-term care management, changes in sick note requirements), recommendations regarding engineering controls (masking, cohorting), and enhanced information needs (antivirals, bed counts).

### Limitations

As with any predictive method, the quality of past performance does not guarantee the quality of future performance. Additionally, the future performance of real-time flu estimates produced with our methodology depends directly on the timely availability and quality of the external data sources used as input. Our findings in the Boston metropolitan area are dependent on Google search volumes, Twitter posts, EHR information, crowd-sourced infection reports, and epidemiological data from the BPHC. Our team’s previous experience nowcasting and forecasting flu activity at the national and regional levels during the past 3 flu seasons has shown us that data availability and acquisition challenges may lead to delays in our flu predictions and may affect the performance of our methods.

### Conclusions

Because transmission happens on a local scale, city-level detection and monitoring can provide useful measures of influenza incidence and risk. Consistent detection on a smaller scale is subject to challenges, such as limitations in data availability; erratic incidence patterns influenced by local factors such as geography, weather, and population movement; and lower signal-to-noise ratio for data sources such as Internet search patterns and crowd-sourced influenza reporting systems. Nevertheless, we show that information from Internet-based data sources, when combined using an informed, robust methodology, can be effectively used as early indicators of flu activity at fine geographic resolution. Successful real-time implementation of an ensemble-based approach to produce robust estimates in the Boston metropolitan area could inform future influenza modeling efforts in other cities.

## Appendix

Multimedia Appendix 1 Comparison between Boston %ILI curve as reported by the Boston Public Health Commission (BPHC) and the US national %ILI as reported by the Centers for Disease Control and Prevention, over the past 5 years. Our best nowcast model for BPHC %ILI is displayed to show strength of fit.

Multimedia Appendix 2 Data processing details for athenahealth and Google Trends.

Multimedia Appendix 3 ARGO model formulation and implementation details.

Multimedia Appendix 4 Details of ensemble methods, including input model layer and comparison of various ensemble predictors.

## Abbreviations

API: application programming interface
ARGO: autoregression with general online information
BPHC: Boston Public Health Commission
CDC: Centers for Disease Control and Prevention
COI: correlation of increment
CORR: Pearson correlation coefficient
ED: emergency department
EHRs: electronic health records
FNY: Flu Near You
GFT: Google Flu Trends
ILI: influenza-like illness
MAE: mean absolute error
MAPE: mean absolute percentage error
RMSE: root mean square error
SIR: susceptible-infected-recovered
