# Supplementary figures and images for: Accurate Influenza Monitoring and Forecasting Using Novel Internet Data Streams: A Case Study in the Boston Metropolis
Source: JMIR Public Health Surveill. 2018 Jan 9;4(1):e4. doi: 10.2196/publichealth.8950 (PMC5780615; doi:10.2196/publichealth.8950)

Comparison of Boston and US National ILI incidence

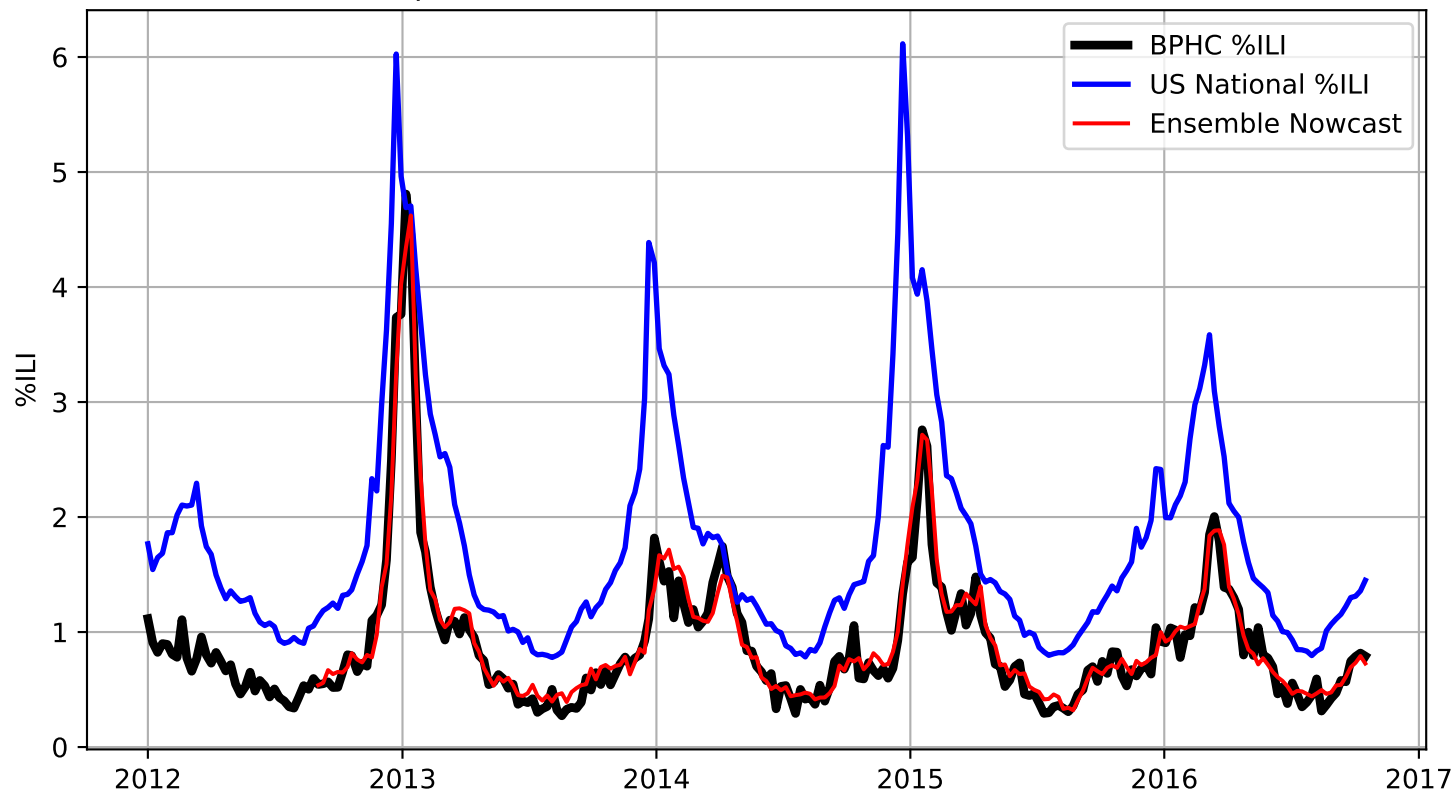

Supplement: Multimedia Appendix 1 [file publichealth_v4i1e4_app1.pdf]
