# Supplementary material for: Accurate Influenza Monitoring and Forecasting Using Novel Internet Data Streams: A Case Study in the Boston Metropolis
Source: JMIR Public Health Surveill. 2018 Jan 9;4(1):e4. doi: 10.2196/publichealth.8950 (PMC5780615; doi:10.2196/publichealth.8950)
Supplement: Multimedia Appendix 2 [file publichealth_v4i1e4_app2.pdf]

1 Google Trends search queries

The following table displays the search queries downloaded from the Google Trends API for our analysis. Sparse search queries (strikethrough) were removed from the predictors.

~~flu incubation~~	~~flu incubation period~~	~~influenza type a~~	symptoms of the flu
flu symptoms	~~influenza symptoms~~	flu contagious	influenza a
a influenza	symptoms of flu	~~flu duration~~	~~influenza incubation~~
~~type a influenza~~	flu treatment	~~symptoms of influenza~~	~~influenza contagious~~
~~flu in children~~	~~cold or flu~~	symptoms of bronchitis	~~flu recovery~~
tessalon	~~influenza incubation period~~	symptoms of pneumonia	tussionex
~~signs of the flu~~	~~flu treatments~~	~~remedies for the flu~~	walking pneumonia
~~flu test~~	tussin	upper respiratory	~~respiratory flu~~
acute bronchitis	bronchitis	sinus infections	~~flu relief~~
~~painful cough~~	~~how long does the flu last~~	flu cough	sinus
expectorant	strep	strep throat	~~influenza treatment~~
~~flu reports~~	~~flu remedy~~	robitussin	~~rapid flu~~
~~treatment for the flu~~	chest cold	cough fever	~~oscillococcinum~~
flu fever	~~treat the flu~~	~~how to treat the flu~~	~~over the counter flu~~
~~how long is the flu~~	flu medicine	~~flu or cold~~	normal body
~~is flu contagious~~	~~treat flu~~	body temperature	reduce fever
~~flu vs cold~~	~~how long is the flu contagious~~	fever reducer	~~get over the flu~~
~~treating flu~~	~~having the flu~~	~~treatment for flu~~	human temperature
~~dangerous fever~~	the flu	~~remedies for flu~~	~~influenza a and b~~
contagious flu	fever flu	~~flu remedies~~	~~how long is flu contagious~~
~~cold vs flu~~	~~braun thermoscan~~	fever cough	~~signs of flu~~
how long does flu last	normal body temperature	~~get rid of the flu~~	~~i have the flu~~
taking temperature	~~flu versus cold~~	how long flu	~~flu germs~~
flu and cold	~~thermoscan~~	~~flu complications~~	high fever
~~flu children~~	~~the flu virus~~	~~how to treat flu~~	pneumonia
~~flu headache~~	ear thermometer	~~how to get rid of the flu~~	flu how long
cold and flu	~~over the counter flu medicine~~	~~treating the flu~~	~~flu care~~
how long contagious	~~fight the flu~~	~~reduce a fever~~	~~cure the flu~~
~~medicine for flu~~	~~flu length~~	~~cure flu~~	~~exposed to flu~~
low body	~~early flu symptoms~~	~~flu report~~	~~incubation period for flu~~
~~break a fever~~	~~flu contagious period~~	~~cold versus flu~~	~~what to do if you have the flu~~
~~medicine for the flu~~	~~flu and fever~~	~~flu lasts~~	~~incubation period for the flu~~
~~do i have the flu~~	boston flu	~~flu in boston~~	massachusetts flu
~~flu in massachusetts~~			

2 Athenahealth processing

Although in previous studies, athenahealth variables were directly divided each week by the 'total patient visit count', we devised a new rate-computing procedure to address the different patient bases between Massachusetts athenahealth providers and the hospitals reporting to the Boston Public Health Commission.

Define $x_{i,t}$ and X_t as the number of athenahealth flu-related visit counts from variable i and total athenahealth visit counts, respectively, in week t. We can assume that $x_{i,t}$ and $X_t - x_{i,t}$ are independent. Similarly, let y_t and Y_t be the BPHC ILI and total patient visit counts respectively, with y_t independent of $Y_t - y_t$. Athenahealth data was traditionally used under the premise that $x_{i,t}/X_t$ is strongly correlated with y_t/Y_t. However, we hypothesize that the distributions of $X_t - x_{i,t}$ and $Y_t - y_t$ are sufficiently independent that $x_{i,t}$ is in fact a better estimator of y_t, compared to $x_{i,t}/X_t$ as an estimator of y_t/Y_t. The justification for this is that athenahealth data generally comes from clinical office-visits all over Massachusetts, whereas the BPHC's ILI rates are computed from emergency department visits within Boston [15]. Thus their respective non-influenza visit counts

each week should generally be uncorrelated. In this context, dividing $x_{i,t}$ by X_t would introduce noise to the athenahealth signal.

Instead of using the raw $x_{i,t}$ as predictors, the three variables were divided with a two-year moving average constructed from the weekly total patient visits to construct smoothed rate variables. Dividing each $x_{i,t}$ by the moving average $\mu(X_{t-104}, X_{t-103}, \ldots, X_t)$ corrects for the gradual increase of cases over time as the athenahealth provider network expanded over the duration of this study.
