# Supplementary material for: Accurate Influenza Monitoring and Forecasting Using Novel Internet Data Streams: A Case Study in the Boston Metropolis
Source: JMIR Public Health Surveill. 2018 Jan 9;4(1):e4. doi: 10.2196/publichealth.8950 (PMC5780615; doi:10.2196/publichealth.8950)
Supplement: Multimedia Appendix 3 [file publichealth_v4i1e4_app3.pdf]

1 ARGO model

1.1 Formulation

ARGO (AutoRegression with General Online information) is a multiple linear regression model combining various sources of information to model influenza activity. The model assumes that at a given time, the %ILI being predicted (which is not yet known) is dependent on the known %ILI from previous weeks, and influences the exogenous data sources which are currently observable. This leads to the hidden Markov model formulation discussed in [30].

ARGO fits variable coefficients by minimizing least squares errors with L1 regularization (LASSO), which leads to the following objective:

$$\sum_t (y_t - \mu_y - \sum_{i=1}^{52} \alpha_i y_{t-i} - \sum_{j \in J} \beta_j X_{j,t} - \sum_{k \in K} \gamma_k W_{k,t})^2 + \lambda_\alpha \sum_{i=1}^{52} |\alpha_i| + \lambda_\beta \sum_{j \in J} |\beta_j| + \lambda_\gamma \sum_{k \in K} |\gamma_k|$$

where y_t is %ILI at time t, X are general online variables including Google Trends and Flu Near You, W are athenahealth variables, μ_y accounts for random noise, and $\lambda_\alpha, \lambda_\beta, \lambda_\gamma$ are regularization hyper-parameters. ARGO is trained using a rolling 104-week window, which ensures that the model dynamically learns from the most recent information and calibrates to changing data patterns.

1.2 Google search transforms

Following the method described in [38], additional modification on Google searches was performed for the ARGO model. The search queries were transformed each week from the following pool of transforms: square-root, square, and log, and none. The version with the highest historical correlation with the official ILI data over the two-year training period was kept. This process dynamically selects, in an out-of-sample process, the transform of each feature that maintains the strongest linear relationship with the target variable.

1.3 Hyper-parameter settings

Reflecting the distinct characteristics of each data source, ARGO incorporates different regularizer weights for auto-regressive terms, Google Trends and other online information, and athenahealth data.

Because athenahealth consistently proved to be the strongest predictor, the regularization on athenahealth variables was decreased to allow their coefficients more weight in the regression. On the other hand, neither of autoregressive or Google Trends terms were more consistently selected by ARGO than the other. Thus we set $\lambda = \lambda_\alpha = \lambda_\beta$, and $\lambda_\gamma/\lambda = r$, where r is 1/5 in the nowcast horizon and 1/25 in the forecast horizon. The constrained regularizers were then determined using 10-fold cross-validation.
