# Supplementary material for: Accurate Influenza Monitoring and Forecasting Using Novel Internet Data Streams: A Case Study in the Boston Metropolis
Source: JMIR Public Health Surveill. 2018 Jan 9;4(1):e4. doi: 10.2196/publichealth.8950 (PMC5780615; doi:10.2196/publichealth.8950)
Supplement: Multimedia Appendix 4 [file publichealth_v4i1e4_app4.pdf]

1 Ensemble

1.1 Input layer

Every non-empty subset of datasets among athena, Google Trends, and AR52 was used as the input for a separate LASSO regression model trained with a sliding two-year window, resulting in 7 models:

AR52: Presented in main text.

athena: The original method in [14] was duplicated, using 9 athenahealth variables with the original rate processing method and 2 autoregressive terms.

Google: The filtered Google searches from the two most recent available weeks were combined as predictors.

athena+Google: The athenahealth and Google Trends variables presented in the main text were combined as predictors.

ARGO(athena): Presented in main text.

ARGO(Google): Presented in main text.

ARGO(athena+Google+FNY): Presented in main text.

1.2 Ensemble models

Predictions were made using all models from the input layer of models. When available, raw FNY and Twitter %ILI data were also included in the models. These predictions were used as the input variables for the following ensemble methods:

Median: The median of the estimates from the input models was used as the ensemble estimate.

Performance-adjusted median: Because of the large performance variance among the input models, we implemented a simple performance-based weighting system. The mean-square error of each individual model with the ILI target over the past 52 weeks was computed, and the poorest-performing model was replaced with a copy of the best-performing model.

Ordinary Least Squares: The individual models were input into an OLS regression with an expanding training window consisting of all data points prior to the prediction week. This essentially provides a performance-adjusted estimate that dynamically weights models based on historical performance.

LASSO: This is an OLS regression with added L1 regularization on the variable coefficients. This improves ensemble predictions by shrinking model coefficients towards 0, effectively deleting individual models with poor historical performance. To further promote solution sparsity, the one-standard-error rule was implemented for regularizer selection and found to improve out-of-sample performance.

Table S1: Comparison of the input layer of models with the Ensemble over the entire test period and each flu season, for the nowcast horizon. The top two models in each category are bolded.

	Whole period (2012-2016)	Flu seasons 2012-2013	2013-2014	2014-2015	2015-2016	*Validation* 2016-2017
RMSE						
AR52	0.303	0.577	0.199	0.305	0.217	0.229
athena	0.208	0.377	**0.163**	0.219	0.144	0.169
Google	0.222	0.218	0.243	0.303	0.221	0.195
athena+Google	0.186	0.210	0.200	0.224	0.194	**0.145**
ARGO(athena)	0.195	0.306	0.229	0.192	**0.133**	0.182
ARGO(Google)	0.206	0.312	0.194	0.247	0.161	0.188
ARGO(athena+Google+FNY)	**0.165**	**0.199**	0.192	**0.189**	0.168	0.156
Ensemble	**0.151**	**0.193**	**0.170**	**0.176**	**0.139**	**0.150**
MAE						
AR52	0.180	0.345	0.146	0.218	0.176	0.176
athena	0.130	0.200	**0.127**	0.172	0.113	0.144
Google	0.169	0.165	0.196	0.241	0.180	0.158
athena+Google	0.144	0.153	0.158	0.187	0.149	**0.115**
ARGO(athena)	0.137	0.205	0.189	**0.136**	**0.102**	0.154
ARGO(Google)	0.150	0.206	0.155	0.213	0.131	0.153
ARGO(athena+Google+FNY)	**0.124**	**0.146**	0.144	0.154	0.128	0.131
Ensemble	**0.112**	**0.140**	**0.131**	**0.135**	**0.106**	**0.118**
MAPE						
AR52	0.184	0.188	0.137	0.185	0.188	0.130
athena	**0.145**	0.124	**0.114**	0.145	0.120	0.118
Google	0.215	0.137	0.192	0.257	0.204	0.130
athena+Google	0.184	**0.112**	0.148	0.195	0.177	**0.090**
ARGO(athena)	0.163	0.128	0.193	**0.124**	**0.110**	0.129
ARGO(Google)	0.179	0.134	0.152	0.209	0.146	0.125
ARGO(athena+Google+FNY)	0.154	**0.112**	0.136	0.153	0.142	0.104
Ensemble	**0.140**	0.100	**0.123**	**0.132**	**0.118**	**0.093**
CORR						
AR52	0.898	0.882	0.846	0.834	0.806	0.898
athena	0.952	0.944	**0.906**	0.952	0.924	0.955
Google	0.947	0.983	0.778	0.875	0.793	0.925
athena+Google	0.964	0.985	0.852	0.955	0.875	**0.959**
ARGO(athena)	0.959	0.964	0.843	0.950	**0.943**	0.949
ARGO(Google)	0.956	0.968	0.856	0.910	0.896	0.930
ARGO(athena+Google+FNY)	**0.972**	**0.985**	0.861	**0.964**	0.916	0.957
Ensemble	**0.976**	**0.986**	**0.890**	**0.964**	**0.928**	**0.958**
COI						
AR52	0.222	0.359	-0.105	0.115	-0.048	0.222
athena	0.463	0.451	**0.465**	**0.642**	0.354	0.367
Google	0.641	**0.821**	**0.501**	0.461	0.278	0.461
athena+Google	0.605	0.790	0.395	0.602	0.221	**0.612**
ARGO(athena)	0.547	0.657	0.220	0.483	**0.486**	0.515
ARGO(Google)	0.546	0.730	0.399	0.284	0.267	0.417
ARGO(athena+Google+FNY)	**0.656**	0.807	0.419	**0.660**	0.312	**0.620**
Ensemble	**0.689**	**0.827**	0.447	0.633	**0.357**	0.565

Table S2: Comparison of the input layer of models with the Ensemble over the entire test period and each flu season, for the forecast horizon. The top two models in each category are bolded.

	Whole period (2012-2016)	Flu seasons 2012-2013	2013-2014	2014-2015	2015-2016	*Validation* 2016-2017
RMSE						
AR52	0.474	0.988	0.246	0.401	0.279	0.354
athena	0.343	0.641	**0.213**	0.404	0.202	**0.233**
Google	0.371	0.683	0.270	0.389	0.235	0.288
athena+Google	0.331	0.642	**0.217**	0.329	0.195	**0.224**
ARGO(athena)	0.325	0.647	0.249	**0.260**	**0.188**	0.261
ARGO(Google)	0.548	1.182	0.329	0.407	0.223	0.290
ARGO(athena+Google+FNY)	**0.245**	**0.367**	0.221	0.314	0.190	0.240
Ensemble	**0.222**	**0.348**	0.237	**0.251**	**0.155**	0.251
MAE						
AR52	0.253	0.559	0.186	0.283	0.211	0.269
athena	0.197	0.394	**0.158**	0.257	0.156	0.192
Google	0.223	0.381	0.215	0.264	0.181	0.222
athena+Google	0.185	0.305	0.174	0.238	0.155	**0.172**
ARGO(athena)	0.203	0.432	0.200	**0.184**	0.156	0.221
ARGO(Google)	0.254	0.549	0.251	0.283	0.174	0.223
ARGO(athena+Google+FNY)	**0.169**	**0.247**	**0.161**	0.225	**0.151**	**0.189**
Ensemble	**0.157**	**0.245**	0.171	**0.202**	**0.123**	0.198
MAPE						
AR52	0.254	0.303	0.167	0.237	0.222	0.193
athena	0.201	0.226	**0.146**	0.214	0.161	0.160
Google	0.256	0.234	0.203	0.273	0.190	0.177
athena+Google	0.208	0.204	0.161	0.220	0.165	**0.132**
ARGO(athena)	0.217	0.254	0.186	**0.168**	0.163	0.175
ARGO(Google)	0.257	0.270	0.229	0.280	0.184	0.177
ARGO(athena+Google+FNY)	**0.192**	**0.167**	**0.142**	0.214	**0.147**	0.149
Ensemble	**0.180**	**0.160**	0.155	**0.198**	**0.130**	**0.144**
CORR						
AR52	0.756	0.679	0.753	0.690	0.673	0.737
athena	0.870	0.829	**0.849**	0.876	0.845	**0.921**
Google	0.873	0.875	0.711	0.777	0.765	0.824
athena+Google	0.912	0.900	0.826	0.890	0.854	0.905
ARGO(athena)	0.887	0.842	0.785	**0.933**	0.898	0.891
ARGO(Google)	0.812	0.798	0.690	0.736	0.796	0.825
ARGO(athena+Google+FNY)	**0.938**	**0.949**	0.826	**0.922**	**0.903**	0.910
Ensemble	**0.944**	**0.956**	**0.847**	0.916	**0.906**	**0.913**
COI						
AR52	0.079	0.116	0.058	-0.061	0.089	-0.112
athena	0.097	-0.052	0.418	0.362	0.063	**0.551**
Google	0.448	**0.625**	0.236	0.241	0.026	0.210
athena+Google	0.401	0.477	**0.490**	0.246	0.280	0.463
ARGO(athena)	0.432	0.452	0.259	**0.624**	**0.312**	0.472
ARGO(Google)	0.352	0.463	0.228	0.125	0.078	0.317
ARGO(athena+Google+FNY)	**0.533**	0.621	**0.451**	**0.508**	0.285	0.477
Ensemble	**0.573**	**0.682**	0.441	0.515	**0.286**	**0.510**

Table S3: Comparison of ensemble models over the entire test period and individual influenza seasons from 2012-2016, for the nowcast horizon. The results from this table were used to select the final ensemble model. The best model in each category is shown in bold.

	Whole period	Flu seasons			
		2012-2013	2013-2014	2014-2015	2015-2016
RMSE					
ARGO(athena+Google+FNY)	0.165	0.199	0.192	0.189	0.168
Median	0.160	0.208	0.176	0.197	0.143
Performance-adjusted median	**0.151**	**0.193**	**0.170**	0.176	**0.139**
OLS	0.183	0.291	0.201	0.270	0.156
LASSO	0.188	0.318	0.195	**0.151**	0.150
MAE					
ARGO(athena+Google+FNY)	0.124	0.146	0.144	0.154	0.128
Median	0.121	0.146	0.141	0.163	0.110
Performance-adjusted median	**0.112**	**0.140**	**0.131**	**0.135**	**0.106**
OLS	0.129	0.193	0.158	0.142	0.121
LASSO	0.125	0.170	0.150	0.139	0.120
MAPE					
ARGO(athena+Google+FNY)	0.154	0.112	0.136	0.153	0.142
Median	0.151	**0.098**	0.134	0.169	0.125
Performance-adjusted median	**0.140**	0.100	**0.123**	**0.132**	**0.118**
OLS	0.150	0.123	0.147	0.145	0.136
LASSO	0.153	0.110	0.140	0.148	0.135
CORR					
ARGO(athena+Google+FNY)	0.972	0.985	0.861	0.964	0.916
Median	0.973	0.983	0.882	0.961	0.924
Performance-adjusted median	**0.976**	**0.986**	**0.890**	0.964	**0.928**
OLS	0.963	0.967	0.849	0.964	0.911
LASSO	0.961	0.964	0.853	**0.965**	0.913
COI					
ARGO(athena+Google+FNY)	0.656	0.807	0.419	**0.660**	0.312
Median	0.658	0.796	0.433	0.579	0.318
Performance-adjusted median	**0.689**	**0.827**	0.447	0.633	**0.357**
OLS	0.551	0.587	**0.516**	0.653	0.353
LASSO	0.477	0.486	0.468	0.650	0.331

Table S4: Comparison of ensemble models over the entire test period and individual influenza seasons from 2012-2016, for the forecast horizon. The results from this table were used to select the final ensemble model. The best model in each category is shown in bold.

		Flu seasons			
	Whole period	2012-2013	2013-2014	2014-2015	2015-2016
RMSE					
ARGO(athena+Google+FNY)	0.245	**0.367**	0.221	0.314	0.190
Median	0.314	0.628	0.209	0.270	0.192
Performance-adjusted median	0.292	0.571	**0.208**	0.258	0.185
OLS	0.319	0.615	0.221	0.316	0.171
LASSO	**0.222**	0.348	0.237	**0.251**	**0.155**
MAE					
ARGO(athena+Google+FNY)	0.169	**0.247**	0.161	0.225	0.151
Median	0.182	0.362	0.154	0.199	0.150
Performance-adjusted median	0.172	0.352	0.149	**0.177**	0.138
OLS	0.182	0.326	0.167	0.220	0.132
LASSO	**0.157**	0.245	**0.171**	0.202	**0.123**
MAPE					
ARGO(athena+Google+FNY)	**0.192**	**0.167**	0.142	0.214	0.147
Median	0.195	0.204	0.136	**0.195**	0.162
Performance-adjusted median	0.184	0.198	0.133	0.162	0.147
OLS	0.199	0.193	0.150	0.200	0.140
LASSO	0.180	0.160	**0.155**	0.198	**0.130**
CORR					
ARGO(athena+Google+FNY)	**0.938**	**0.949**	0.826	**0.922**	**0.903**
Median	0.895	0.855	0.832	0.905	0.855
Performance-adjusted median	0.906	0.875	0.830	0.907	0.870
OLS	0.897	0.864	0.820	0.915	0.888
LASSO	0.936	0.937	**0.836**	**0.922**	0.900
COI					
ARGO(athena+Google+FNY)	**0.533**	**0.621**	0.451	0.508	0.285
Median	0.360	0.418	0.371	0.295	0.184
Performance-adjusted median	0.431	0.523	0.287	0.385	0.166
OLS	0.272	0.268	0.405	0.444	**0.292**
LASSO	0.506	0.567	**0.477**	**0.527**	0.272
